# Supplementary material for: Multiple cardiovascular risk factor care in 55 low- and middle-income countries: A cross-sectional analysis of nationally-representative, individual-level data from 280,783 adults
Source: PLOS Glob Public Health. 2024 Mar 27;4(3):e0003019. doi: 10.1371/journal.pgph.0003019 (PMC10971750; doi:10.1371/journal.pgph.0003019)
Supplement: S1 Table — (DOCX) [file pgph.0003019.s001.docx]

**Multiple Cardiovascular Risk Factor Care in 55 Low- and Middle-Income Countries Supplement Material**

**S1 Table.** Detailed definitions the blood pressure and diabetes biomarker measurements

| **Population definition** | **Definitions** |
| --- | --- |
| Hypertension criteria and blood pressure measurement | Hypertension was defined as meeting any one of the following criteria: systolic blood pressure of at least 140 mm Hg or diastolic blood pressure of at least 90 mmHg, reported antihypertensive medication use, or reported diagnosis by a clinician.  There were one to three blood pressure measurements collected for each participant during the survey visit. For participants with three blood pressure measurements, we used the mean of the last two measurements to reduce the white coat effect—a change in BP levels due to the presence of a physician or other health professional [1]. For participants with only two measurements, we computed the mean of both available measurements. |
| Diabetes criteria and diabetes biomarker measurements | Diabetes was defined as meeting any one of the following criteria: fasting plasma glucose ≥7.0 mmol/L (126 mg/dL), random plasma glucose ≥11.1 mmol/L (200mg/dL), hemoglobin A1c ≥6.5%, reported glucose-lowering medication use, or reported diagnosis by a clinician.  The diabetes biomarker used for diagnosis was a point-of-care fasting capillary glucose in 41 surveys, a laboratory-based fasting plasma glucose in seven surveys, and hemoglobin A1c in eight surveys. In countries with capillary glucose measurements, we converted values to plasma glucose by multiplying by a factor of 1.11 based on research showing that capillary glucose underestimates plasma concentrations. Where fasting status was missing, with one exception, we assumed that the glucose measurement was fasting in accordance with survey protocols. The exception was India where random blood glucose was the primary diabetes biomarker [2]. |

*Abbreviation*: mg/dl, milligram/deciliter; mmHg, millimeter of mercury; mmol/L, millimole/liter; WHO PEN, WHO Package of Essential Noncommunicable Disease Interventions for Primary Health Care in Low-resource Settings.

References

1. Pioli MR, Ritter AM, de Faria AP, Modolo R. White coat syndrome and its variations: differences and clinical impact. *Integr Blood Press Control*. 2018;11:73-79. doi:10.2147/IBPC.S152761

2. Flood D, Seiglie JA, Dunn M, et al. The state of diabetes treatment coverage in 55 low-income and middle-income countries: a cross-sectional study of nationally representative, individual-level data in 680 102 adults. *The Lancet Healthy Longevity*. May 2021. doi:10.1016/S2666-7568(21)00089-17. WHO CVD Risk Chart Working Group. World Health Organization cardiovascular disease risk charts: revised models to estimate risk in 21 global regions. *Lancet Glob Health*. 2019;7(10):e1332-e1345. doi:10.1016/S2214-109X(19)30318-3
