# Supplementary material for: Multiple cardiovascular risk factor care in 55 low- and middle-income countries: A cross-sectional analysis of nationally-representative, individual-level data from 280,783 adults
Source: PLOS Glob Public Health. 2024 Mar 27;4(3):e0003019. doi: 10.1371/journal.pgph.0003019 (PMC10971750; doi:10.1371/journal.pgph.0003019)
Supplement: S2 Table — (DOCX) [file pgph.0003019.s002.docx]

| S2 Table. Blood pressure measurement details | | | |
| --- | --- | --- | --- |
| Country | Measurement device | Number of measurements | Interval between measurements |
| Algeria | Not specified | 3 | Not specified |
| Azerbaijan | Not specified | 3 | Not specified |
| Bangladesh | Boso-Medicus Control (universal cuff) | 3 | 3 minutes |
| Belarus | Boso-Medicus Uno | 3 | 3 minutes |
| Benin | Boso-Medicus Uno | 3 | 3 minutes |
| Bhutan | Boso-Medicus Control | 3 | 3 minutes |
| Botswana | Not specified | 3 | Not specified |
| Burkina Faso | Omron Digital Monitor HEM­705CP | 3 | 10 minutes |
| Cambodia | NISSEI Digital Blood Pressure Monitor (Model DS-500) | 3 | Not specified |
| Chile | Omron Digital Monitor HEM­742 | 3 | 2 minutes |
| Comoros | Digital upper arm meter (model not specified) | 2 | 5-10 minutes |
| Costa Rica | Digital upper arm meter (Welch Allyn and Omron, models not specified) | 2 with a third measurement if the first two differed by >10 mmHg | 5 minutes |
| Ecuador | Digital upper arm meter (model not specified) | 2 with a third measurement if the first two differed by >5 mmHg | 5 minutes |
| Eritrea | Omron M7 Digital Monitor | 2 with a third measurement if the first two differed by >10 mmHg | Not specified |
| Eswatini | Boso Medicus PC (model not specified) | 3 | 3-5 minutes |
| Georgia | Boso-Medicus Uno | 3 | 3 minutes |
| Guyana | Omron digital upper arm meter (model not specified) | 3 | 3 minutes |
| India | Rossmax AW150 | 2 | 10 minutes |
| Indonesia | Omron Digital Monitor HEM­7203 | 3 | First measurement taken at beginning of interview, subsequent two taken during the course of the interview |
|  |  |  |  |
| Iran | Beurer medical, type: BM 20 | 3 | 5 minutes |
| Iraq | Manual mercury sphygmomanometer | 3 | Not specified |
| Kenya | Omron M2 Digital Monitor | 3 | 3-5 minutes |
| Kyrgyzstan | Not specified | 3 | Not specified |
| Laos | Omron | 3 | Not specified |
| Lebanon | Omron M6 comfort | 3 | Not specified |
| Lesotho | Omron M3W Digital Monitor | 3 | 10 minutes |
| Liberia | Omron digital upper arm meter (model not specified) | 3 | Not specified |
| Marshall Islands | Not specified | 3 | Not specified |
| Mexico | Omron digital upper arm meter (model not specified) | 2 | Not specified |
| Moldova | Boso-Medicus Uno | 3 | 3 minutes |
| Mongolia | Omron M5 Digital Monitor | 3 | 3 minutes |
| Morocco | Spengler ES 60 | 3 | Not specified |
| Myanmar | Boso-Medicus Uno | 3 | 3-5 minutes |
| Namibia | Life Source Digital  Monitor Model UA-767 | 3 | Not specified |
| Nepal | Omron digital upper arm meter (model not specified) | 3 | 3 minutes |
| Romania | Automatic Device, A&D UA 95 Plus | 3 | 1 minute |
| Rwanda | Omron M4 Digital Automatic Monitor | 3 | 2-3 minutes |
| Samoa | Omron Digital Automatic Monitor | 2 with a third measurement if the first two differed by >10 mmHg | Not specified |
| São Tomé and Principe | Unable to identify | Unable to identify | Unable to identify |
| Seychelles | Omron M3 Digital Monitor | 5 | Once upon arrival, three times during the interview, and once before leaving |
| Solomon Islands | Omron M4 Digital Automatic Monitor | 3 | 2-3 minutes |
| Sri Lanka | Not specified | 3 | Not specified |
| Sudan | Not specified | 3 | Not specified |
| St. Vincent and the Grenadines | Omron Digital Monitor M4 - I | 3 | 3 minutes |
| Tajikistan | Not specified | 3 | Not specified |
| Tanzania | Omron digital upper arm meter (model not specified) | 3 | Not specified |
| Timor-Leste | Omron digital upper arm meter (model not specified) | 3 | 2 minutes |
| Togo | Omron digital upper arm meter (model not specified) | 3 | 5 minutes |
| Tuvalu | Unable to identify | 3 | Unable to identify |
| Uganda | Boso Medicus Uno | 3 | 3-5 minutes |
| Vanuatu | Omron M4 | 3 | 2-3 minutes |
| Vietnam | Boso Device (model not specified) | Not specified | Not specified |
| Zambia | Automated Blood Pressure Monitor | 3 | 3-5 minutes |
| Zanzibar | Omron M2 Digital Monitor | 3 | 5 minutes |
