# Supplementary material for: Multiple cardiovascular risk factor care in 55 low- and middle-income countries: A cross-sectional analysis of nationally-representative, individual-level data from 280,783 adults
Source: PLOS Glob Public Health. 2024 Mar 27;4(3):e0003019. doi: 10.1371/journal.pgph.0003019 (PMC10971750; doi:10.1371/journal.pgph.0003019)
Supplement: S3 Table — (DOCX) [file pgph.0003019.s003.docx]

**S3 Table. Diabetes biomarker measurement details**

| **Diabetes Biomarker** | **Country** | **Post Hoc Adjustment*** |
| --- | --- | --- |
| *Point-of-care fasting capillary glucose* |  |  |
| Accu-check | Samoa, Tuvalu | None |
| Accutrend® Plus (Roche, Basel, Switzerland) | Cambodia, Chile, Guyana, Liberia, São Tomé and Principe, Togo, Zanzibar | Multiplied by 1.11 |
| CardioCheck® PA (pts Diagnostics, Indianapolis, Indiana, USA) | Belarus, Benin, Bhutan, Burkina Faso, Eswatini, Kenya, Kiribati,Moldova, Morocco, Nepal, Rwanda, Solomon Islands, Sri Lanka, St. Vincent & The Grenadines, Timor-Leste, Sudan, Uganda, Vietnam, Zambia | None |
| CONTOUR® (Ascensia Diabetes Care Holdings AG, Basel, Switzerland) | Seychelles | None |
| FreeStyle Optium H glucometer | India | Multiplied by 1.11 |
| HemoCue® Glucose 201 Analyzer (HemoCue, Brea, California, USA) | Namibia, Tanzania | None |
| MultiCare-in© (Biochemical Systems International, Arezzo, Italy) | Georgia | None |
| SD LipidoCare Analyzer (automatic plasma equivalent) | Myanmar | None |
| Prima home test | Mongolia | None |
| Unknown | Algeria, Azerbaijan, Botswana, Comoros, Ecuador, Eritrea, Kyrgyzstan, Laos, Lesotho, Marshall Islands, Tajikistan, Vanuatu | None |
| *Laboratory-based Assessment of Fasting Plasma Glucose* | | |
| Auto analyzer Selectrao Pro M Human ®, Germany | Bangladesh | N/A |
| Central laboratory was used for processing | Lebanon | N/A |
| Cobas 6000 and C311 analyzer (Roche Diagnostics, Indianapolis, Indiana, USA) | Iran, Romania | N/A |
| Enzymatic assay (glucose oxidase) | Iraq | N/A |
| SYNCHRON® System (Beckman Coulter, Inc., Miami, Florida, USA) | Costa Rica | N/A |
| *Hemoglobin A1c (HbA1c)* |  | N/A |
| Point-of-care Bayer Consumer Care AG (A1cNow) | Seychelles |  |
| Dried blood spots using the Hemocue system | Indonesia | N/A |
| Plasma sample by Cobas C311 auto-analyzer (Roche kits) | Iran | N/A |
| Point-of-care In2ItTM device by Bio-Rad | Mexico | N/A |
| Unknown | Guyana | N/A |
| Venous blood Cobas 6000 | Romania |  |
