# Supplementary material for: Multiple cardiovascular risk factor care in 55 low- and middle-income countries: A cross-sectional analysis of nationally-representative, individual-level data from 280,783 adults
Source: PLOS Glob Public Health. 2024 Mar 27;4(3):e0003019. doi: 10.1371/journal.pgph.0003019 (PMC10971750; doi:10.1371/journal.pgph.0003019)
Supplement: S4 Table — (DOCX) [file pgph.0003019.s004.docx]

**S4 Table. Lipid biomarker measurement details**

In seven countries biomarkers were measured via blood samples sent to a laboratory; in 22 countries biomarkers were measured with a point-of-care device; in six countries, the biomarker measurement method could not be identified.

| **Measurement** | **Country** |
| --- | --- |
| Accutrend GCT | Eritrea |
| Accutrend Plus | Cambodia, Togo, Tuvalu, Zanzibar |
| Accutrend (model not specified) | São Tomé and Principe, Tanzania, Vanuatu |
| Accuchek | Samoa |
| CardioCheck PA | Belarus, Benin, Bhutan, Burkina Faso, Ecuador, Eswatini, Kenya, Kiribati, Laos, Lesotho, Mexico, Moldova, Morocco, Rwanda, Solomon Islands, Sri Lanka, St. Vincent & the Grenadines, Sudan, Timor-Leste, Uganda, Vietnam, Zambia |
| Konelab 30i | Seychelles |
| Laboratory | Bangladesh, Chile, Costa Rica, Guyana, Iran, Iraq, Lebanon, Romania |
| Prima Home Test | Mongolia |
| SD LipidoCare Analyzer | Myanmar |
| Unknown | Algeria, Azerbaijan, Botswana, Georgia, Kyrgyzstan, Marshall Islands, Tajikistan |
