# Supplementary material for: Multiple cardiovascular risk factor care in 55 low- and middle-income countries: A cross-sectional analysis of nationally-representative, individual-level data from 280,783 adults
Source: PLOS Glob Public Health. 2024 Mar 27;4(3):e0003019. doi: 10.1371/journal.pgph.0003019 (PMC10971750; doi:10.1371/journal.pgph.0003019)
Supplement: S5 Table — (DOCX) [file pgph.0003019.s005.docx]

**S5 Table.** Select questions from generic STEPS surveys

The generic versions of the World Health Organization STEPwise approach to noncommunicable disease surveillance (WHO STEPS) instrument are available online (accessed June 26, 2020):

Version 2.1: <https://www.who.int/ncds/surveillance/steps/STEPS_Instrument_v2.1.pdf>

Version 3.2: <https://www.who.int/ncds/surveillance/steps/instrument/STEPS_Instrument_V3.2.pdf>

Select questions used to generate healthcare service coverage indicators are described below.

| **Indicator** | **Version 2.1** | **Version 3.2** |
| --- | --- | --- |
| 1. Diagnosis awareness | Have you ever been told by a doctor or other health worker that you have raised blood pressure [blood glucose] or hypertension [diabetes]? [Yes/No] | Have you ever been told by a doctor or other health worker that you have raised blood pressure [blood glucose] or hypertension [diabetes]? [Yes/No] |
| 1. Counseling to start or increase exercise | Are you currently receiving any of the following treatments/advice for high blood pressure [blood glucose] prescribed by a doctor or other health worker?  Advice to start or do more exercise [Yes/No] | During any of your visits to a doctor or other health worker in the past 12 months, were you advised to do any of the following?  Start or do more physical activity [Yes/No] |
| 1. Counseling on salt reduction | Are you currently receiving any of the following treatments/advice for high blood pressure prescribed by a doctor or other health worker?  Advise to reduce salt intake [Yes/No] | During any of your visits to a doctor or other health worker in the past 12 months, were you advised to do any of the following?  Reduce salt in your diet [Yes/No] |
| 1. Counseling on weight-loss | Are you currently receiving any of the following treatments/advice for diabetes prescribed by a doctor or other health worker?  Advise or treatment to lease weight [Yes/No] | During any of your visits to a doctor or other health worker in the past 12 months, were you advised to do any of the following?  Maintain a healthy body weight or lose weight [Yes/No] |
| 1. Antihypertensive medication | Are you currently receiving any of the following treatments/advice for high blood pressure prescribed by a doctor or other health worker?  Drugs (medication) that you have taken in the past two weeks [Yes/No] | In the past two weeks, have you taken any drugs (medication) for raised blood pressure prescribed by a doctor or other health worker? [Yes/No] |
| 1. Glucose-lowering medication | Diabetes medication:  Are you currently receiving any of the following treatments/advice for diabetes prescribed by a doctor or other health worker?  Drugs (medication) that you have taken in the past two weeks [Yes/No]  Insulin use:  Are you currently receiving any of the following treatments/advice for diabetes prescribed by a doctor or other health worker?  Insulin [Yes/No] | Diabetes medication:  In the past two weeks, have you taken any drugs (medication) for diabetes prescribed by a doctor or other health worker? [Yes/No]  Insulin use:  Are you currently taking insulin for diabetes prescribed by a doctor or other health worker? [Yes/No] |
| 1. Cholesterol-lowering medication | Cholesterol medication use  During the past two weeks, have you been treated for raised cholesterol with drugs (medication) prescribed by  a doctor or other health worker? [Yes/No] | Cholesterol medication use  During the past two weeks, have you been treated for raised cholesterol with drugs (medication) prescribed by a doctor or other health worker? [Yes/No] |

*Note*: There was variability between generic survey versions with respect to the availability, phrasing, and skip patterns for the involved questions. For example, the “Lifestyle Advice” section is a core module in version 3, whereas in version 2 lifestyle counseling questions were only asked to respondents reporting a previous diagnosis of diabetes or hypertension. In addition, there is a small degree of variability in the way countries have chosen to implement questions in their surveys.
