# Supplementary material for: Multiple cardiovascular risk factor care in 55 low- and middle-income countries: A cross-sectional analysis of nationally-representative, individual-level data from 280,783 adults
Source: PLOS Glob Public Health. 2024 Mar 27;4(3):e0003019. doi: 10.1371/journal.pgph.0003019 (PMC10971750; doi:10.1371/journal.pgph.0003019)
Supplement: S6 Table — (DOCX) [file pgph.0003019.s006.docx]

**S6 Table.** Number and percent of participants with missing characteristics variables by country*

| **Geographic region and country** | **Age** | **Sex** | **Education** | **Body mass index** | **Current tobacco smoke** |
| --- | --- | --- | --- | --- | --- |
|  | N (%) | | | | |
| ***East, South, and Southeast Asia*** |  |  |  |  |  |
| Bangladesh | 0 (0.0) | 0 (0.0) | 1 (0.1) | 2 (0.1) | 0 (0.0) |
| Bhutan | 0 (0.0) | 0 (0.0) | 1 (0.1) | 3 (0.4) | 0 (0.0) |
| Cambodia | 0 (0.0) | 0 (0.0) | 0 (0.0) | 4 (0.6) | 0 (0.0) |
| India | 0 (0.0) | 0 (0.0) | 0 (0.0) | 107 (0.2) | 0 (0.0) |
| Indonesia | 0 (0.0) | 0 (0.0) | 13 (1.0) | 16 (1.2) | 0 (0.0) |
| Laos | 0 (0.0) | 0 (0.0) | 0 (0.0) | 2 (0.6) | 0 (0.0) |
| Myanmar | 0 (0.0) | 0 (0.0) | 8 (0.3) | 5 (0.2) | 1 (0.0) |
| Nepal | 0 (0.0) | 0 (0.0) | 1 (0.1) | 2 (0.2) | 0 (0.0) |
| Sri Lanka | 0 (0.0) | 0 (0.0) | 6 (0.5) | 12 (0.9) | 0 (0.0) |
| Timor-Leste | 0 (0.0) | 0 (0.0) | 13 (2.8) | 3 (0.6) | 0 (0.0) |
| Vietnam | 0 (0.0) | 0 (0.0) | 0 (0.0) | 3 (0.4) | 1 (0.1) |
| ***Europe and Central Asia*** |  |  |  |  |  |
| Azerbaijan | 0 (0.0) | 0 (0.0) | 2 (0.2) | 37 (3.9) | 0 (0.0) |
| Belarus | 0 (0.0) | 0 (0.0) | 0 (0.0) | 4 (0.2) | 0 (0.0) |
| Georgia | 0 (0.0) | 0 (0.0) | 76 (5.4) | 15 (1.1) | 0 (0.0) |
| Kyrgyzstan | 0 (0.0) | 0 (0.0) | 0 (0.0) | 1 (0.1) | 0 (0.0) |
| Moldova | 0 (0.0) | 0 (0.0) | 1 (0.1) | 24 (1.5) | 0 (0.0) |
| Mongolia | 0 (0.0) | 0 (0.0) | 0 (0.0) | 4 (0.7) | 0 (0.0) |
| Romania | 0 (0.0) | 0 (0.0) | 33 (7.2) | 0 (0.0) | 33 (7.2) |
| Tajikistan | 0 (0.0) | 0 (0.0) | 1 (0.1) | 2 (0.2) | 0 (0.0) |
| ***Latin America and the Caribbean*** |  |  |  |  |  |
| Chile | 0 (0.0) | 0 (0.0) | 3 (0.3) | 14 (1.3) | 3 (0.3) |
| Costa Rica | 0 (0.0) | 1 (0.1) | 37 (5.2) | 52 (7.3) | 0 (0.0) |
| Ecuador | 0 (0.0) | 0 (0.0) | 0 (0.0) | 5 (0.6) | 0 (0.0) |
| Guyana | 0 (0.0) | 0 (0.0) | 2 (0.8) | 1 (0.4) | 0 (0.0) |
| Mexico | 0 (0.0) | 0 (0.0) | 103 (3.5) | 195 (6.6) | 299 (10.2) |
| St. Vincent & the Grenadines | 0 (0.0) | 0 (0.0) | 0 (0.0) | 1 (0.3) | 0 (0.0) |
| ***Middle East and North Africa*** |  |  |  |  |  |
| Algeria | 0 (0.0) | 0 (0.0) | 8 (0.5) | 6 (0.4) | 3 (0.2) |
| Iran | 0 (0.0) | 0 (0.0) | 138 (3.1) | 49 (1.1) | 21 (0.5) |
| Iraq | 0 (0.0) | 0 (0.0) | 2 (0.2) | 13 (1.1) | 0 (0.0) |
| Lebanon | 0 (0.0) | 0 (0.0) | 14 (3.6) | 0 (0.0) | 0 (0.0) |
| Morocco | 0 (0.0) | 0 (0.0) | 1 (0.1) | 6 (0.5) | 0 (0.0) |
| ***Oceania*** |  |  |  |  |  |
| Kiribati | 0 (0.0) | 0 (0.0) | 7 (2.2) | 7 (2.2) | 1 (0.3) |
| Marshall Islands | 0 (0.0) | 0 (0.0) | 1 (0.1) | 30 (4.4) | 7 (1.0) |
| Samoa | 0 (0.0) | 0 (0.0) | 7 (1.8) | 0 (0.0) | 0 (0.0) |
| Solomon Islands | 0 (0.0) | 0 (0.0) | 2 (0.6) | 4 (1.2) | 1 (0.3) |
| Tuvalu | 0 (0.0) | 0 (0.0) | 2 (0.5) | 11 (2.9) | 0 (0.0) |
| Vanuatu | 0 (0.0) | 0 (0.0) | 9 (0.8) | 11 (0.9) | 0 (0.0) |
| ***Sub-Saharan Africa*** |  |  |  |  |  |
| Benin | 0 (0.0) | 0 (0.0) | 1 (0.1) | 0 (0.0) | 0 (0.0) |
| Botswana | 0 (0.0) | 0 (0.0) | 0 (0.0) | 6 (0.8) | 0 (0.0) |
| Burkina Faso | 0 (0.0) | 0 (0.0) | 0 (0.0) | 0 (0.0) | 0 (0.0) |
| Comoros | 0 (0.0) | 0 (0.0) | 1 (0.2) | 22 (3.9) | 0 (0.0) |
| Eritrea | 0 (0.0) | 0 (0.0) | 5 (0.7) | 1 (0.1) | 0 (0.0) |
| Eswatini | 0 (0.0) | 0 (0.0) | 0 (0.0) | 12 (2.1) | 0 (0.0) |
| Kenya | 0 (0.0) | 0 (0.0) | 0 (0.0) | 6 (0.8) | 1 (0.1) |
| Lesotho | 0 (0.0) | 0 (0.0) | 1 (0.2) | 8 (1.3) | 2 (0.3) |
| Liberia | 0 (0.0) | 0 (0.0) | 8 (2.7) | 8 (2.7) | 0 (0.0) |
| Namibia | 0 (0.0) | 0 (0.0) | 8 (0.6) | 16 (1.3) | 37 (3.0) |
| Rwanda | 0 (0.0) | 0 (0.0) | 0 (0.0) | 0 (0.0) | 0 (0.0) |
| São Tomé and Principe | 0 (0.0) | 0 (0.0) | 4 (0.8) | 15 (2.9) | 1 (0.2) |
| Seychelles | 0 (0.0) | 0 (0.0) | 0 (0.0) | 1 (0.2) | 0 (0.0) |
| Sudan | 0 (0.0) | 0 (0.0) | 3 (0.2) | 10 (0.6) | 0 (0.0) |
| Tanzania | 0 (0.0) | 0 (0.0) | 0 (0.0) | 3 (0.3) | 0 (0.0) |
| Togo | 0 (0.0) | 0 (0.0) | 0 (0.0) | 1 (0.2) | 1 (0.2) |
| Uganda | 0 (0.0) | 0 (0.0) | 5 (1.1) | 7 (1.5) | 0 (0.0) |
| Zambia | 0 (0.0) | 0 (0.0) | 1 (0.2) | 0 (0.0) | 0 (0.0) |
| Zanzibar | 0 (0.0) | 0 (0.0) | 0 (0.0) | 5 (0.8) | 0 (0.0) |
| Global | 0 (0.0) | 1 (0.0) | 579 (0.5) | 831 (0.7) | 425 (0.4) |
