# Supplementary material for: Multiple cardiovascular risk factor care in 55 low- and middle-income countries: A cross-sectional analysis of nationally-representative, individual-level data from 280,783 adults
Source: PLOS Glob Public Health. 2024 Mar 27;4(3):e0003019. doi: 10.1371/journal.pgph.0003019 (PMC10971750; doi:10.1371/journal.pgph.0003019)
Supplement: S7 Table — (DOCX) [file pgph.0003019.s007.docx]

**S7 Table.** Number and percent of participants with missing outcome indicator variables by country

| **Geographic region and country** | **Diagnosed: Hypertension** | **Diagnosed: Diabetes** | **Exercise counseling** | **Weight-loss counseling** | **Salt-reduction counseling*** | **Antihypertensive medication** | **Glucose-lowering medication** | **Cholesterol-lowering medication** | **BP target** | **BG target** |
| --- | --- | --- | --- | --- | --- | --- | --- | --- | --- | --- |
|  | **N (%)** | | | | | | | | | |
| ***East, South, and Southeast Asia*** |  |  |  |  |  |  |  |  |  |  |
| Bangladesh | 0 (0.0) | 0 (0.0) | 0 (0.0) | 0 (0.0) | 0 (0.0) | 0 (0.0) | 0 (0.0) | 0 (0.0) | 0 (0.0) | 0 (0.0) |
| Bhutan | 0 (0.0) | 0 (0.0) | 0 (0.0) | 0 (0.0) | 0 (0.0) | 0 (0.0) | 0 (0.0) | 0 (0.0) | 0 (0.0) | 0 (0.0) |
| Cambodia | 0 (0.0) | 0 (0.0) | N/A | N/A | N/A | 0 (0.0) | 0 (0.0) | N/A | 0 (0.0) | 0 (0.0) |
| India | 3 (0.0) | 0 (0.0) | N/A | N/A | N/A | 3 (0.0) | 0 (0.0) | N/A | 0 (0.0) | 10,993 (93.2) |
| Indonesia | 7 (0.6) | 2 (0.7) | N/A | N/A | N/A | 7 (0.6) | 2 (0.7) | N/A | 0 (0.0) | 0 (0.0) |
| Laos | 0 (0.0) | 0 (0.0) | N/A | N/A | N/A | 0 (0.0) | 0 (0.0) | N/A | 0 (0.0) | 0 (0.0) |
| Myanmar | 1 (0.0) | 0 (0.0) | 1 (0.0) | 0 (0.0) | 1 (0.0) | 1 (0.0) | 0 (0.0) | 0 (0.0) | 0 (0.0) | 0 (0.0) |
| Nepal | 0 (0.0) | 0 (0.0) | 0 (0.0) | 0 (0.0) | 0 (0.0) | 0 (0.0) | 0 (0.0) | 0 (0.0) | 0 (0.0) | 0 (0.0) |
| Sri Lanka | 1 (0.1) | 0 (0.0) | 1 (0.1) | 0 (0.0) | 1 (0.1) | 1 (0.1) | 0 (0.0) | 0 (0.0) | 0 (0.0) | 1 (0.2) |
| Timor-Leste | 0 (0.0) | 0 (0.0) | 1 (0.2) | 0 (0.0) | 1 (0.2) | 0 (0.0) | 0 (0.0) | 0 (0.0) | 0 (0.0) | 0 (0.0) |
| Vietnam | 1 (0.2) | 0 (0.0) | 3 (0.4) | 1 (1.0) | 2 (0.3) | 1 (0.2) | 0 (0.0) | 2 (2.1) | 0 (0.0) | 7 (7.3) |
| ***Europe and Central Asia*** |  |  |  |  |  |  |  |  |  |  |
| Azerbaijan | 0 (0.0) | 0 (0.0) | N/A | N/A | N/A | 0 (0.0) | 0 (0.0) | 0 (0.0) | 0 (0.0) | 0 (0.0) |
| Belarus | 0 (0.0) | 0 (0.0) | 0 (0.0) | 0 (0.0) | 0 (0.0) | 0 (0.0) | 0 (0.0) | 0 (0.0) | 0 (0.0) | 0 (0.0) |
| Georgia | 0 (0.0) | 0 (0.0) | 0 (0.0) | 0 (0.0) | 0 (0.0) | 0 (0.0) | 0 (0.0) | 0 (0.0) | 0 (0.0) | 0 (0.0) |
| Kyrgyzstan | 0 (0.0) | 0 (0.0) | 0 (0.0) | 0 (0.0) | 0 (0.0) | 0 (0.0) | 0 (0.0) | 0 (0.0) | 0 (0.0) | 0 (0.0) |
| Moldova | 5 (0.3) | 0 (0.0) | 5 (0.3) | 0 (0.0) | 5 (0.3) | 5 (0.3) | 0 (0.0) | 0 (0.0) | 0 (0.0) | 0 (0.0) |
| Mongolia | 0 (0.0) | 0 (0.0) | 0 (0.0) | 0 (0.0) | 0 (0.0) | 0 (0.0) | 0 (0.0) | 0 (0.0) | 0 (0.0) | 0 (0.0) |
| Romania | 0 (0.0) | 0 (0.0) | N/A | N/A | N/A | 0 (0.0) | 0 (0.0) | 0 (0.0) | 0 (0.0) | 0 (0.0) |
| Tajikistan | 0 (0.0) | 0 (0.0) | 0 (0.0) | 0 (0.0) | 0 (0.0) | 0 (0.0) | 0 (0.0) | 0 (0.0) | 0 (0.0) | N/A |
| ***Latin America and the Caribbean*** |  |  |  |  |  |  |  |  |  |  |
| Chile | 2 (0.2) | 2 (0.6) | N/A | N/A | N/A | 2 (0.2) | 2 (0.6) | N/A | 0 (0.0) | 15 (4.2) |
| Costa Rica | 0 (0.0) | 0 (0.0) | N/A | 9 (3.8) | N/A | 0 (0.0) | 0 (0.0) | N/A | 0 (0.0) | N/A |
| Ecuador | 0 (0.0) | 0 (0.0) | 0 (0.0) | 0 (0.0) | 0 (0.0) | 0 (0.0) | 0 (0.0) | 0 (0.0) | 0 (0.0) | 0 (0.0) |
| Guyana | 0 (0.0) | 0 (0.0) | 0 (0.0) | 0 (0.0) | 0 (0.0) | 0 (0.0) | 0 (0.0) | 0 (0.0) | 0 (0.0) | 8 (7.8) |
| Mexico | 61 (3.1) | 71 (4.0) | N/A | N/A | N/A | 61 (3.1) | 71 (4.0) | N/A | 0 (0.0) | 0 (0.0) |
| St. Vincent & the Grenadines | 0 (0.0) | 0 (0.0) | 0 (0.0) | 0 (0.0) | 0 (0.0) | 0 (0.0) | 0 (0.0) | 0 (0.0) | 0 (0.0) | 5 (4.9) |
| ***Middle East and North Africa*** |  |  |  |  |  |  |  |  |  |  |
| Algeria | 2 (0.1) | 3 (0.5) | 4 (0.2) | 3 (0.5) | 2 (0.1) | 2 (0.1) | 3 (0.5) | 3 (0.5) | 0 (0.0) | 6 (1.0) |
| Iran | 12 (0.3) | 2 (0.2) | 0 (0.0) | 0 (0.0) | 0 (0.0) | 12 (0.3) | 2 (0.2) | 2 (0.2) | 0 (0.0) | 13 (1.0) |
| Iraq | 75 (7.1) | 71 (14.7) | 0 (0.0) | 0 (0.0) | 1 (0.1) | 75 (7.1) | 71 (14.7) | 0 (0.0) | 0 (0.0) | 0 (0.0) |
| Lebanon | 0 (0.0) | 0 (0.0) | 0 (0.0) | 0 (0.0) | 0 (0.0) | 0 (0.0) | 0 (0.0) | 0 (0.0) | 0 (0.0) | 0 (0.0) |
| Morocco | 0 (0.0) | 0 (0.0) | 0 (0.0) | 0 (0.0) | 0 (0.0) | 0 (0.0) | 0 (0.0) | 0 (0.0) | 0 (0.0) | 0 (0.0) |
| ***Oceania*** |  |  |  |  |  |  |  |  |  |  |
| Kiribati | 2 (0.8) | 0 (0.0) | 2 (0.6) | 0 (0.0) | 2 (0.8) | 2 (0.8) | 0 (0.0) | 0 (0.0) | 0 (0.0) | 0 (0.0) |
| Marshall Islands | 0 (0.0) | 0 (0.0) | N/A | N/A | N/A | 0 (0.0) | 0 (0.0) | N/A | 0 (0.0) | 0 (0.0) |
| Samoa | 0 (0.0) | 0 (0.0) | N/A | N/A | N/A | 0 (0.0) | 0 (0.0) | N/A | 0 (0.0) | 0 (0.0) |
| Solomon Islands | 1 (0.3) | 1 (1.5) | 2 (0.6) | 1 (1.5) | 1 (0.3) | 1 (0.3) | 1 (1.5) | 1 (1.5) | 0 (0.0) | 0 (0.0) |
| Tuvalu | 0 (0.0) | 0 (0.0) | 0 (0.0) | 0 (0.0) | 0 (0.0) | 0 (0.0) | 0 (0.0) | 0 (0.0) | 0 (0.0) | 0 (0.0) |
| Vanuatu | 0 (0.0) | 2 (0.4) | N/A | N/A | N/A | 0 (0.0) | 2 (0.4) | N/A | 0 (0.0) | 9 (1.8) |
| ***Sub-Saharan Africa*** |  |  |  |  |  |  |  |  |  |  |
| Benin | 0 (0.0) | 0 (0.0) | 0 (0.0) | 0 (0.0) | 0 (0.0) | 0 (0.0) | 0 (0.0) | 0 (0.0) | 0 (0.0) | 0 (0.0) |
| Botswana | 0 (0.0) | 0 (0.0) | 0 (0.0) | 0 (0.0) | 0 (0.0) | 0 (0.0) | 0 (0.0) | 0 (0.0) | 0 (0.0) | 0 (0.0) |
| Burkina Faso | 44 (10.3) | 0 (0.0) | 0 (0.0) | 0 (0.0) | 0 (0.0) | 44 (10.3) | 0 (0.0) | 0 (0.0) | 0 (0.0) | 0 (0.0) |
| Comoros | 0 (0.0) | 0 (0.0) | N/A | N/A | N/A | 0 (0.0) | 0 (0.0) | N/A | 0 (0.0) | 0 (0.0) |
| Eritrea | 0 (0.0) | 0 (0.0) | N/A | N/A | N/A | 0 (0.0) | 0 (0.0) | N/A | 0 (0.0) | 0 (0.0) |
| Eswatini | 0 (0.0) | 0 (0.0) | 0 (0.0) | 0 (0.0) | 0 (0.0) | 0 (0.0) | 0 (0.0) | 0 (0.0) | 0 (0.0) | 0 (0.0) |
| Kenya | 1 (0.1) | 0 (0.0) | 0 (0.0) | 0 (0.0) | 0 (0.0) | 1 (0.1) | 0 (0.0) | 0 (0.0) | 0 (0.0) | 1 (1.3) |
| Lesotho | 2 (0.3) | 0 (0.0) | N/A | N/A | N/A | 2 (0.3) | 0 (0.0) | N/A | 0 (0.0) | 0 (0.0) |
| Liberia | 0 (0.0) | 0 (0.0) | N/A | N/A | N/A | 0 (0.0) | 0 (0.0) | N/A | 0 (0.0) | 0 (0.0) |
| Namibia | 2 (0.2) | 0 (0.0) | N/A | N/A | N/A | 2 (0.2) | 0 (0.0) | N/A | 0 (0.0) | 0 (0.0) |
| Rwanda | 0 (0.0) | 0 (0.0) | N/A | N/A | N/A | 0 (0.0) | 0 (0.0) | N/A | 0 (0.0) | 0 (0.0) |
| São Tomé and Principe | 0 (0.0) | 0 (0.0) | N/A | N/A | N/A | 0 (0.0) | 0 (0.0) | N/A | 0 (0.0) | 16 (51.6) |
| Seychelles | 0 (0.0) | 0 (0.0) | 0 (0.0) | 0 (0.0) | 0 (0.0) | 0 (0.0) | 0 (0.0) | N/A | 0 (0.0) | 0 (0.0) |
| Sudan | 0 (0.0) | 0 (0.0) | 0 (0.0) | 0 (0.0) | 0 (0.0) | 0 (0.0) | 0 (0.0) | 0 (0.0) | 0 (0.0) | 0 (0.0) |
| Tanzania | 0 (0.0) | 0 (0.0) | N/A | N/A | N/A | 0 (0.0) | 0 (0.0) | N/A | 0 (0.0) | 0 (0.0) |
| Togo | 1 (0.3) | 1 (2.0) | N/A | N/A | N/A | 1 (0.3) | 1 (2.0) | N/A | 0 (0.0) | 0 (0.0) |
| Uganda | 0 (0.0) | 0 (0.0) | 0 (0.0) | 0 (0.0) | 0 (0.0) | 0 (0.0) | 0 (0.0) | 0 (0.0) | 0 (0.0) | 0 (0.0) |
| Zambia | 0 (0.0) | 0 (0.0) | 0 (0.0) | 0 (0.0) | 0 (0.0) | 0 (0.0) | 0 (0.0) | 0 (0.0) | 0 (0.0) | 0 (0.0) |
| Zanzibar | 0 (0.0) | 0 (0.0) | N/A | N/A | N/A | 0 (0.0) | 0 (0.0) | N/A | 0 (0.0) | 0 (0.0) |

*Abbreviation*: N/A, not applicable—outcome indicator was not measured in this survey.
