# Supplementary material for: Multiple cardiovascular risk factor care in 55 low- and middle-income countries: A cross-sectional analysis of nationally-representative, individual-level data from 280,783 adults
Source: PLOS Glob Public Health. 2024 Mar 27;4(3):e0003019. doi: 10.1371/journal.pgph.0003019 (PMC10971750; doi:10.1371/journal.pgph.0003019)
Supplement: S8 Table — (DOCX) [file pgph.0003019.s008.docx]

**S8 Table.** Distribution of 10-year predicted cardiovascular disease (CVD) risk score among individuals with hypertension only, diabetes only and hypertension and diabetes (hypertension-diabetes)

| Calculator  *Restriction* | Study groups | CVD risk score  %–(95% CI) | |
| --- | --- | --- | --- |
|  |  | <10% | ≥10% |
| Office  *None*  *55 countries* | Hypertension | 37.9 (36.7–39.1) | 62.1 (60.9–63.3) |
|  | Diabetes | 62.2 (59.8–64.6) | 37.8 (35.4–40.2) |
|  | Hypertension-Diabetes | 72.4 (70.4–74.3) | 72.4 (70.4–74.3) |
|  |  |  |  |
| Office  *Lipid data collected*  *34 countries* | Hypertension | 36.5 (35.2–37.8) | 63.5 (62.2–64.8) |
|  | Diabetes | 61.0 (58.3–63.7) | 39.0 (36.3–41.7) |
|  | Hypertension-Diabetes | 26.4 (24.4–28.5) | 73.6 (71.5–75.6) |
|  |  |  |  |
| Laboratory  *Lipid data collected*  *34 countries* | Hypertension | 49.0 (47.6–50.4) | 51.0 (49.6–52.4) |
|  | Diabetes | 34.1 (31.7–36.6) | 65.9 (63.4–68.3) |
|  | Hypertension-Diabetes | 12.6 (11.2–13.9) | 87.4 (86.1–88.8) |
