# Supplementary material for: Multiple cardiovascular risk factor care in 55 low- and middle-income countries: A cross-sectional analysis of nationally-representative, individual-level data from 280,783 adults
Source: PLOS Glob Public Health. 2024 Mar 27;4(3):e0003019. doi: 10.1371/journal.pgph.0003019 (PMC10971750; doi:10.1371/journal.pgph.0003019)
Supplement: S9 Table — (DOCX) [file pgph.0003019.s009.docx]

**S9 Table.** The proportion of individuals who were aware of their condition (diagnosed), had received lifestyle counseling, used pharmacological therapy, and achieved appropriate management for cardiovascular disease risk reduction among those with hypertension (HTN) only, diabetes (DM) only, and hypertension and diabetes (HTN-DM) in 56 low- and middle-income countries overall and by world bank income group.

| Outcomes  *Indicator* | Study groups^a^ | Overall | Low/lower-middle income countries | Upper-middle income countries | 2009–2014 | 2015–2019 |
| --- | --- | --- | --- | --- | --- | --- |
|  |  | Percentage (95% CI)^b^ | | | | |
| Awareness of condition |  |  |  |  |  |  |
| *Diagnosed* | HTN | 47.4 (45.3–49.6) | 40.8 (39.9–41.8) | 61.0 (56.4–65.5) | 45.8 (44.2– 47.4) | 49.6 (45.2–54.0) |
|  | DM | 46.7 (44.1–49.2) | 38.5 (35.6–41.4) | 56.2 (50.3–62.0) | 37.7 (34.8–40.6) | 54.9 (49.9–59.9) |
|  | HTN-DM | 64.1 (61.8–66.4) | 56.6 (54.0–59.3) | 72.2 (68.2–76.3) | 62.7 (60.2– 65.3) | 65.3 (61.5–69.1) |
| Lifestyle counseling |  |  |  |  |  |  |
| *Exercise* | HTN | 39.7 (38.3–41.0) | 35.9 (34.4–37.5) | 47.2 (43.9–50.4) | 41.9 (39.9–43.9) | 38.0 (36.1–40.0) |
|  | DM | 42.3 (38.9–45.7) | 33.4 (29.8–37.0) | 54.3 (48.1–60.5) | 42.4 (36.7–48.1) | 42.3 (38.1–46.4) |
|  | HTN-DM | 55.8 (52.1–59.5) | 50.2 (45.0–55.4) | 62.4 (57.9–67.0) | 61.0 (56.8–65.1) | 53.2 (48.0–58.4) |
|  |  |  |  |  |  |  |
| *Weight-loss* | HTN | 36.6 (35.4–37.8) | 32.0 (30.6–33.3) | 45.3 (42.6–47.9) | 38.2 (36.5–39.9) | 35.4 (33.6–37.1) |
|  | DM | 37.5 (34.3–40.6) | 29.2 (25.6–32.8) | 48.1 (42.8–53.5) | 39.0 (33.4–44.6) | 36.8 (33.0–40.7) |
|  | HTN-DM | 54.2 (50.8–57.7) | 47.3 (42.3–52.3) | 61.9 (58.1–65.7) | 58.3 (55.1–61.5) | 51.9 (46.8–57.0) |
|  |  |  |  |  |  |  |
| *Salt reduction* | HTN | 46.2 (44.7–47.6) | 43.6 (42.0–45.2) | 51.3 (47.6–54.9) | 51.2 (48.3–54.0) | 42.5 (40.6–44.4) |
|  | HTN-DM | 59.9 (56.5–63.3) | 56.1 (51.1–61.0) | 64.5 (60.3–68.8) | 65.6 (60.4–70.8) | 57.1 (52.6–61.5) |
| Pharmacological therapy |  |  |  |  |  |  |
| *Antihypertensive* | HTN | 20.9 (19.6–22.3) | 17.2 (16.5–18.0) | 28.6 (23.3–33.8) | 20.3 (18.6– 22.1) | 21.7 (19.4–24.0) |
|  | HTN-DM | 38.3 (34.8–41.8) | 32.7 (29.8–35.6) | 44.5 (36.9–52.2) | 39.9 (34.8–44.9) | 36.8 (32.1–41.5) |
|  |  |  |  |  |  |  |
| *Glucose-lowering* | DM | 29.3 (27.2–31.4) | 23.0 (20.4–25.6) | 36.6 (31.0–42.1) | 24.5 (22.0–26.9) | 33.7 (29.4–38.0) |
|  | HTN-DM | 42.3 (39.4–45.2) | 32.9 (30.4–35.4) | 52.6 (46.3–58.9) | 41.7 (38.3–45.1) | 42.8 (38.2–47.4) |
|  |  |  |  |  |  |  |
| *Combined antihypertensive and glucose-lowering* | HTN-DM | 24.6 (22.1–27.2) | 17.3 (15.4–19.2) | 32.6 (26.5–38.8) | 24.8 (21.1–28.6) | 24.4 (20.9–27.9) |
|  |  |  |  |  |  |  |
| *Statins* | DM | 4.6 (3.5–5.6) | 3.3 (2.4–4.3) | 6.2 (4.1–8.2) | 4.4 (2.6–6.3) | 4.6 (3.3–5.9) |
|  | HTN-DM | 9.5 (8.3–10.7) | 6.7 (5.5–7.9) | 12.6 (10.3–15.0) | 9.1 (6.9–11.3) | 9.7 (8.2–11.1) |
| Appropriate management |  |  |  |  |  |  |
| *Blood pressure (BP)* | HTN | 17.6 (16.4–18.8) | 13.6 (12.9–14.3) | 25.9 (23.7–28.2) | 17.3 (16.1– 18.6) | 18.0 (15.7–20.2) |
|  | HTN-DM | 24.6 (22.5–26.6) | 18.1 (16.1–20.1) | 31.8 (28.8–34.8) | 26.3 (23.4–29.1) | 23.0 (20.1–26.0) |
|  |  |  |  |  |  |  |
| *Blood glucose (BG)* | DM | 13.3 (10.7–15.8) | 10.3 (8.6–12.1) | 20.8 (13.3–28.3) | 12.1 (9.6– 14.6) | 15.0 (10.0–20.0) |
|  | HTN-DM | 20.0 (17.8–22.3) | 16.0 (13.6–18.3) | 27.9 (23.7–32.0) | 19.8 (16.6– 22.9) | 20.4 (17.1–23.7) |
|  |  |  |  |  |  |  |
| *Combined BP and BG* | HTN-DM | 6.6 (5.4–7.8) | 4.7 (3.4–5.9) | 10.3 (8.2–12.4) | 6.1 (4.4–7.8) | 7.3 (5.7–8.9) |
|  |  |  |  |  |  |  |
| *Combined BP and BG plus statin use* | DM | 1.4 (0.8–1.9) | 1.1 (0.5–1.7) | 2.0 (0.9–3.1) | 1.7 (0.6–2.7) | 1.2 (0.6–1.8) |
|  | HTN-DM | 1.6 (1.1–2.0) | 0.9 (0.6–1.3) | 2.6 (1.6–3.6) | 0.9 (0.3–1.5) | 2.0 (1.3–2.6) |

^a^We identified three, mutually-exclusive study groups. Individuals with hypertension only were those with blood pressure ≥140/90 mmHg or self-reported antihypertensive medication use or self-reported diagnosis by a clinician, without the diabetes. Individuals with diabetes only were those with a fasting plasma glucose of 7.0 mmol/L (126 mg/dL) or above, random plasma glucose 11.1 mmol/L (200mg/dL) or above, an HbA1c measurement of 6.5% or above or self-reporting using glucose-lowering medications or self-reported diagnosis by a clinician, without the hypertension. Individuals with hypertension and diabetes were those with concurrent hypertension and diabetes, and they were our primary study population.

^b^The percentage of eligible individuals who received a healthcare service and achieved appropriate management for cardiovascular disease risk reduction. Eligibility is based on the World Health Organization (WHO) Package of Essential Noncommunicable (PEN) Disease Interventions to frame our evaluation [1] according to the conditions.

References

1. 1. World Health Organization. *WHO package of essential noncommunicable (PEN) disease interventions for primary health care*. Geneva: World Health Organization; 2020.
