# Supplementary material for: Multiple cardiovascular risk factor care in 55 low- and middle-income countries: A cross-sectional analysis of nationally-representative, individual-level data from 280,783 adults
Source: PLOS Glob Public Health. 2024 Mar 27;4(3):e0003019. doi: 10.1371/journal.pgph.0003019 (PMC10971750; doi:10.1371/journal.pgph.0003019)
Supplement: Attachment — Submitted filename: HPACC-MM_PLOS-GPH_RandR-letter-submit.docx [file pgph.0003019.s010.docx]

**Dear Dr. Ziyue Wang,**

Thank you for your positive review of our manuscript. On behalf of my co-authors, I am happy to submit this revision for further consideration. Below we provide a point-by-point response to the reviewers’ and editor’s suggestions.

We look forward to your review.

Sincerely,

Emily Gower

**Response to Editors and Reviewers**

**Reviewer 1, comment 1:  My primary concern is around the framing and scientific and policy relevance of the paper. Papers documenting the hypertension cascade (regardless of diabetes) and the diabetes cascade (regardless of hypertension) are valuable for revealing gaps and focusing policy attention. However, the main contribution of this paper is the focus on those that are both hypertensive and diabetic. It isn't clear to me what additional insights we gain from the authors' analysis of this population that we wouldn't have gotten from focusing on just those with hypertension or just those with diabetes. Put differently, if there are existing policies focused on hypertension improvement and diabetes improvement, wouldn't these also catch those with both conditions? I struggle to think of how we might especially miss those with both conditions.

One suggestion to the authors for increasing the relevance of the paper might be to focus on "missed opportunities" for diagnosis or treatment. For example, what share of those with both hypertension and diabetes are diagnosed or taking treatment for just one of the two conditions (e.g. what proportion of those with both are diagnosed for hypertension but not diabetes or taking treatment for diabetes but not hypertension)? Such discrepancies - if they exist in the data - have more direct policy relevance as they suggest that policies and care focused purely on hypertension are missing out on the opportunity to diagnose and treat a significant share of people with diabetes (and potentially vice versa). To play devil's advocate, if most people with hypertension don't have diabetes, then maybe it is actually more cost-effective to just focus on hypertension (hopefully your data shows that this isn't the case).**

We appreciate the reviewer’s concern regarding the scientific and policy relevance of the paper. For scientific relevance, we state in the introduction that “Coexisting CVD risk factors (e.g., hypertension, diabetes, hyperlipidemia) increase the complexity of symptom management needed to prevent further deterioration of quality of life and health [5,6], contributing to increased morbidity and mortality [7,8].” Thus, using existing single-disease prevention and management policies may not resolve challenges arising from interactions among these conditions at the pathophysiological, socioeconomic, and health systems levels.

For policy relevance, we have added a paragraph to the introduction section on the ratification of the global diabetes coverage targets by the 74^th^ World Health Assembly in 2022. These targets emphasize the need to prevent and manage diabetes and its complications and comorbidities (e.g., hypertension and hyperlipidemia) concurrently via a comprehensive CVD care approach as part of larger efforts to reach global sustainable development goals (SDG) target 3.4, which calls for a reduction in premature mortality from non-communicable diseases (NCDs) by a third by 2030 relative to 2015 levels. As part of this ratification, it was recommended to increase the health system’s capacity to monitor progress toward achieving the global diabetes coverage targets, and our results will serve as baseline data for evaluating progress toward these targets. This paragraph can be found on pages 6–7.

“The 75th World Health Assembly (2022) ratified global coverage targets for diabetes to prevent diabetes-related complications as part of high-level efforts to reach global sustainable development goals (SDG) target 3.4, which calls for a reduction in premature mortality from non-communicable diseases (NCDs) by a third by 2030 relative to 2015 levels, through diabetes prevention and management [14]. Global coverage targets were established for diabetes because it is a major obstacle to achieving SDG target 3.4 given that it is strongly associated with other CVD risk factors like hypertension and hyperlipidemia and is a leading cause of mortality [15–17]. The global diabetes coverage targets are that by 2030, 80% of people living with diabetes are clinically diagnosed; and among them, 80% have good control of glycemia (hemoglobin A1c <8%), and 80% have good control of blood pressure (<140/90 mm Hg), and 60% of those with diabetes aged 40 years or older are taking a statin; 100% of those with type 1 diabetes have access to affordable insulin and blood glucose self-monitoring [14]. These targets highlight the increasing recognition that CVD risk factors including hypertension, diabetes, and hyperlipidemia should be managed concurrently. Additionally, the Assembly agreed on recommendations to increase health systems' capacity to deliver cost-effective population-wide interventions and monitor progress towards these targets.”

Additionally, we presented results on “missed opportunities” throughout the results section and discussed them in the discussion section on pages 21–23. For example, we state on page 22:

“Here, we found greater use of pharmacological therapy for one condition in individuals with hypertension-diabetes compared to those who had either condition alone, which suggests that people with both conditions are interacting with the health system and provided more opportunities to receive needed care.”

**Reviewer 1, comment 2: My second concern with the paper is around the multivariable prediction analyses. As it is written, this set of analyses isn't motivated and seems almost mechanical, as if it is something everyone has to do in papers (I am personally guilty of this as well). I would encourage the authors to motivate these analyses better and tailor the models to the motivation. If the goal is to identify disparities, then simple stratified estimates/bivariate regressions would be the appropriate way (since the real world is not adjusted). If the goal is to develop a prediction model that could be used by practitioners, then the authors might consider predictive accuracy as the goal and compare different model approaches to predicting. As it stands, a multivariable regression of different social and demographic variables does satisfyingly answer either motivation.**

Thank you for your suggestion. We have dropped this objective and the accompanying contents/paragraphs in the abstract, introduction, methods, and results sections. We did not discuss the results in the discussion section.

**Reviewer 2, comment 1: How to ensure national representativeness when different countries have different population sizes and different sample sizes included in the analysis, for example, China, where just over 500 participants were included in the analysis.**

Thank you for your question. Each survey used a representative sampling frame with varying sample sizes across surveys. The analytical sample size differed from the survey sample size after we applied our study inclusion criteria (non-pregnant individuals aged 40–69 years with physiological measures of blood pressure and either blood glucose or hemoglobin A1c). We described our rationale and approach on page 11.

“In all analyses, we accounted for the complex survey design by adjusting for stratification and clustering at the primary sampling unit (PSU) using the ‘srvyr’ R package [34]. Additionally, we used sampling weights adjusting for selection probability, nonresponse, and differences between the sample and target population. The main interest of our analysis is at the health system level; therefore, we rescaled survey weights to ensure equal contribution of each survey.”

Regarding the 2009 China Health and Nutrition Survey (CHNS), there were 16,837 respondents, and only 539 (3.2%) met the inclusion criteria. The CHNS was conducted in eight provinces—Liaoning, Shandong, Henan, Jiangsu, Hubei, Hunan, Guizhou, and Guangxi (from north to south), and is thus not nationally representative. We have dropped the CHNS for our analysis and updated the results in the manuscript and supplemental materials. Excluding the CHNS did not change our conclusions. We reviewed documentation on the remaining 55 surveys used in our analysis and confirmed that these were nationally representative.

**CHNS reference:** Popkin BM, Du S, Zhai F, Zhang B. Cohort Profile: The China Health and Nutrition Survey--monitoring and understanding socio-economic and health change in China, 1989-2011. Int J Epidemiol. 2010;39: 1435–1440. doi:10.1093/ije/dyp322

**Reviewer 2, comment 2: The surveys included in the study span a relatively long period of time, how to eliminate the time factor from interfering with the results?**

We thank you for your question. To explore the potential impact of time on the results arising from how surveys implemented the same question and blood pressure and glucose were measured across time and country, we have now included stratified results in the supplemental materials comparing findings from surveys implemented during 2009–2014 and 2015–2019. Although we preferred to examine outcomes by year, we selected these two time periods to ensure a sufficient sample size for each study group (hypertension only, diabetes only, and both hypertension and diabetes). The stratified results are presented in (S9 Table). We did not observe statistically significant differences in outcomes (diagnosis, treatment, and appropriate management) between surveys conducted during 2009–2014 and 2015–2019. Additionally, we include extensive information on the underlying surveys in our supplemental materials. We have included the following language in the results:

“We did not observe statistically significant differences in outcomes (diagnosis, treatment, and appropriate management) between surveys conducted during 2009–2014 and 2015–2019 (S9 Table).”
